# Effects of Calving Body Condition Score on Blood Acid–Base Balance of Primiparous Holstein-Friesian Dairy Cows in a Commercial Dairy Farm: A Case Study

**DOI:** 10.3390/ani11072075

**Published:** 2021-07-13

**Authors:** Rodrigo Muiño, Joaquín Hernández, José L. Benedito, Cristina Castillo

**Affiliations:** Animal Pathology Department, Veterinary School, University of Santiago de Compostela, Campus of Lugo, 27002 Lugo, Spain; rodrigomuino@colvet.es (R.M.); joseluis.benedito@usc.es (J.L.B.); cristina.castillo@usc.es (C.C.)

**Keywords:** heifers, parturition, body condition score, nutrition, acid–base balance, milk yield

## Abstract

**Simple Summary:**

This study assesses the effect of the body condition score (BCS) on productive variables and the acid–base balance in Holstein heifers. For this purpose, two groups were established according to the BCS: a group of 12 animals with an optimal BCS (range 3.25–3.5) and a group of 15 heifers with a BCS higher than 3.5. The study period started one month before calving (BC) and ran until one month after calving (AC). The results showed that the BCS of heifers does not affect milk production in terms of quantity and quality. In addition, the study of acid–base balance variables provides information that contributes additional insights into metabolic changes that can sometimes go unnoticed.

**Abstract:**

The study was carried out on 27 healthy primiparous Holstein heifers (620 ± 50 kg) kept in a commercial dairy herd. The animals were divided into two groups taking into account the body condition score (BCS) index: BCS < 3.5, *n* = 12; BCS > 3.5 *n* = 15. The study period started one month before calving (BC), and ran until one month after calving (AC). Venous blood samples were collected 1 month and 1 week BC, and 1 week and 1 month AC. This study had two objectives: (i) to assess whether a higher or lower BCS affected total milk production and its quality; (ii) to assess changes in the internal fluid (venous pH; partial pressure of CO_2_, ppCO_2_; bicarbonate; total CO_2_, TCO_2_; base excess, BE; electrolytes Na^+^, K^+^, Cl^−^; and anion gap, AG) that occur during this phase depending on the BCS. We can conclude that the BCS at calving does not affect the productive status during lactation, both in terms of the quantity and quality of milk produced. The excess of crude protein (CP) added through the ration in the lactation phase can trigger a tendency to an alkalotic state, in this case compensated by respiratory buffering mechanisms, as reflected by the TCO_2_. The changes in electrolytes are a reflection of the movement of free water for milk production, where a balance between measurable anions and cations is observed.

## 1. Introduction

On a dairy farm, reproductive efficiency determines, to a large extent, the farm’s profits in terms of milk production and successive pregnancies. This reproductive efficiency depends on many factors, such as the health status of the animal and the diet it receives. The Holstein breed has been selected to produce high yields at the expense of a greater propensity for losing body condition in early lactation due to a higher milk production and the absorption of nutrients preferentially by the mammary gland [1].

In general, many authors suggest that the beginning of a heifer’s reproductive lifespan should be around 23–25 months [2,3] since this implies a decrease in production costs. Moreover, the same studies that recommend this age also emphasize the fact that the heifer is still in the growth phase, so nutritional and management requirements must be taken into account when compared to multiparous dairy cows. However, the reality is that this is a management decision peculiar to each farm.

In any case, the studies that were consulted refer to the age of the first calving as a determining factor in the incidence of numerous postpartum pathological processes, both metabolic and reproductive, which often lead the heifer to its culling [4].

Biochemical biomarkers have been used to confirm the metabolic changes that occur during calving, especially in the transition period. This stage has been widely studied, although there are still aspects to be solved. A recent review [5] extensively describes the endocrine-metabolic and inflammatory changes that take place during this critical phase, pointing to the existence of what is called metabolic stress. This situation has been chiefly based on studies on multiparous cows and biochemical parameters, especially those related to oxidative stress, the immune system or metabolic profiles. However, the parameters of acid–base balance are rarely included [6], even though blood pH is a reflection of the changes that occur in the internal balance, including changes in compensatory mechanisms. This lack of information may be due partly to the fact that these variables are more expensive to monitor than others, such as the body condition score (BCS) or milk production.

Indeed, a commonly used parameter has been that of the BCS as a predictor of the greater or lesser risk that a heifer may develop a more or less marked state of negative energy balance (NEB) after birth. It is recommended for all ages, that the BCS should not exceed 3.5 [7,8,9] according to the well-known Edmonson scale [10]. It has been reported that a high BCS and greater decline of BCS are related to high lipid mobilization and, possibly, to the incidence of metabolic disorders such as ketosis [7] in the transition period.

In accordance with the above and taking into account the scarcity of studies on the changes in the acid–base balance in heifers at calving, this study has two objectives: (1) to assess whether a higher or lower BCS affects total milk production and its quality; (2) to assess the changes in the internal fluid (venous pH; partial pressure of CO_2_, ppCO_2_; bicarbonate; Total CO_2_, TCO_2_; Base excess, BE; electrolytes Na^+^, K^+^, Cl^−^, and anion gap, AG) that occur during this phase depending on the BCS.

We would like to emphasize that this study was conducted in a commercial dairy farm, with heifers with a highly variable BCS ranging from 3.2 to 4.5, and in a different environment from those conducted in experimental facilities with standard management and feeding conditions. We hope that this study is of some help to veterinarians who may find themselves in similar situations since, as Cooke et al. [3] indicate, farmers need such information to make management decisions on heifer growth rates and breeding strategies.

## 2. Materials and Methods

The study was carried out on 27 healthy primiparous Holstein heifers (620 ± 50 kg) at an age of 25 ± 2 months; kept in a commercial dairy herd located in Lugo (Galicia, North-West Spain). The sample size calculation was calculated using G*Power power analysis software, based on a predesigned effect size of a small difference between the groups, based on Cohen’s principles [11].

The animals were divided into two groups taking into account the BCS index which was recorded according to the Edmonson scale [10] and where the value of 1 indicates severe under-conditioning and 5 is severe over-conditioning. This assessment was carried out by the same person. In this way, prior to the first sampling, animals were classified into two groups: in one group we included those heifers with a body condition considered adequate for the month prior to parturition (3.25–3.5, BCS ≤ 3.5 group, mean body weight 550 ± 10 kg) and the other group was composed of those heifers with a BCS higher than 3.5, even reaching 4.5 (BCS > 3.5 group, mean body weight 600 ± 10 kg). It is noteworthy that from this first measurement, the heifers gained around 0.8 ± 0.1 kg/day. Thus, the number of animals per group was 12 heifers in the BCS < 3.5 group and 15 in the BCS > 3.5 group.

The study period started one month before calving (BC) and ran until one month after calving (AC) with a variation between samplings of 2–3 days for the animals included in each group. All heifers had a normal, easy calving (unassisted or assisted by one person) and no clinical abnormalities were seen during the postpartum period. The calving season was in autumn, between 10 and 30 October 2019. During the experimental period, all the animals were kept under identical conditions.

The diet for heifers in the prior parturition period, based on the National Research Council (NRC, 2001), consisted of a base ration, fed as a daily total mixed ration (TMR) comprising corn silage (4 kg), wheat straw (6 kg) and 5.2 kg of a commercial concentrate composed of rapeseed meal, barley and beetroot molasses. After calving, the diet was adjusted to maintain the requirements of milk output. The postpartum diet consisted of corn silage (35 kg), grass silage (6 kg) and 12.6 kg of a specific concentrate composed of corn, rapeseed meal, soybean flour, barley and beetroot molasses. The chemical composition of both diets is shown in Table 1.

The average dry matter intake (DMI, kg/d) was calculated by weighing feed refusals of concentrate and straw daily at 08:00 before feeding. Thus, DMI was 11 ± 1 for BCS-3.5 and 10 ± 1 kg for BCS > 3.5 before parturition. After calving, the average DMI (kg/d) was 22.5 ± 0.5 for BCS-3.5 and 21 ± 0.5 for BCS > 3.5.

All cows were housed in a free-stall barn bedded with wood shavings. The diet was offered ad libitum as a total mixed ration three times daily at 07:00, 14:00, and 20:00 h. The experiment conducted in this report was approved by the Animal Care and Use Committee of the University of Santiago de Compostela, according to the Spanish Regulations (RD 53/2013, legal provision number 1337) and the European regulation of animals for scientific purposes (Council of Europe, ETS no.123) [12].

Venous blood samples were collected via jugular puncture at 1 month (sampling 1) and 1 week (sampling 2) before the expected calving, and at 1 week and 1 month after parturition (samplings 3 and 4, respectively). In all of the cases, samples were collected following the second meal, between 15:00 and 16:00 h.

Blood acid–base parameters and electrolytes (pH; partial pressure of CO_2_, ppCO_2_; Bicarbonate HCO_3_^−^; Base excess, BE; Total CO_2_, TCO_2_; Na^+^; K^+^; Cl^−^; and anion gap, AG) were determined using whole blood samples drawn anaerobically from the jugular vein and measured immediately using a hand-held portable analyzer (i-STAT EC8+, East Windsor, NJ, USA). Physiological and pathological control blood samples were analyzed alongside the samples to provide a two-point quality control.

Samples of TMR were collected at the beginning of each stage and submitted for chemical analysis to the laboratory of the commercial dairy farm. Analytical procedures were as follows: European Union standard methods were used for starch (European Directive 99/79/EC, 1999). Both neutral detergent fiber (NDF) and acid detergent fiber (ADF) were analyzed according to the method of Van Soest et al. [13] with amylase and sodium sulfite and expressed exclusive of residual ash. Nitrogen content was determined using the combustion method according to the Dumas principle, described by the French Association of Standardization [14].

Milk collection was carried out by the Friesian Breeders Association of Lugo (AFRICOR) in the same week as sampling. This legally authorized company recorded the daily yields of the animals previously identified with the farm code, the identification code of the bovine, and the calving date. A milk sample from each collection was sent to the Official Dairy Analysis Laboratory of Galicia, which, using official techniques based on molecular spectroscopy techniques, determined the physicochemical composition of the raw milk (fat, proteins) as well as the number of somatic cells present in the milk (by flow cytometry). The fat/protein ratio (F/P ratio), also known as the ketosis index, was calculated from the fat and protein values. Duffield [15] and Richardt [16] defined an F/P ratio value of 1.5 as a risk level for subclinical ketosis. Since milk fat and milk protein percentages are altered in subclinical ketosis, these parameters have been investigated for their utility in defining subclinical ketosis in this study.

The somatic cell count was log transformed into a somatic cell concentration (SCCn) by the formula SCCn = log 2 (SCC/100,000) + 3 [17].

The data were analyzed by a Mixed Linear Effects Model with the Ime4 package [18] in R statistical packages, version 4.0.3 [19] to evaluate whether the sampling moment and BCS status modified the acid–base and milk yield parameters. The model included sampling (T-effect: one week and one month before calving, BC; one week and one month after calving, AC); and BCS (TR-effect: BCS-3.5 group and 15 BCS > 3.5 group) as fixed factors, and the animal as a repeated (random) factor. The dependent variables were milk production, fat, protein, fat/protein ratio (F/P ratio), SCC count and acid–base parameters. The *p*-values for the mixed model were obtained with the ImerTest package. The descriptive values were obtained from the analysis with the emmeans (Estimated marginal Means) function. Significance was declared at *p*-values < 0.05. Post-hoc pair analyses were performed by Tukey’s honestly significant difference (HSD) test.

## 3. Results

**Figure 1:**
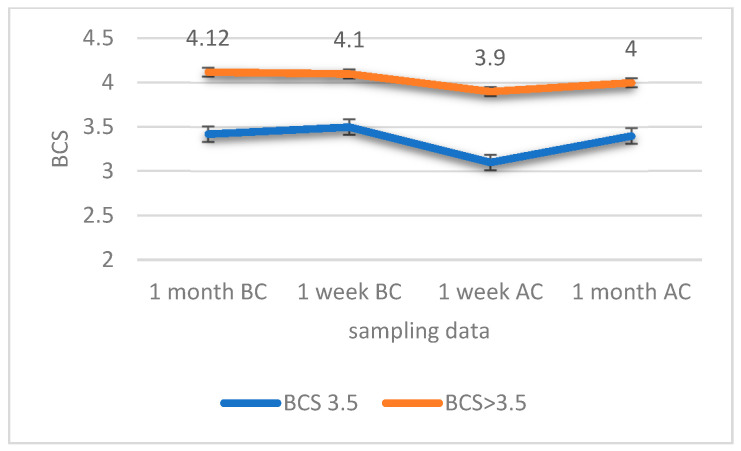
Body condition score (BCS) depending on the time of sampling. Standard error of the mean (SEM) for each group/sampling was 0.13; 0.0; 0.34 and 0.14 for BCS-3.5 heifers, whereas for the BCS > 3.5 group they were 0.3; 0.3; 0.12; and 0.3 for 1 month before calving (BC); 1 week BC; 1 week after calving (AC) and 1 month AC, respectively.

Figure 1 shows the evolution of BCS in both groups throughout the study. Time (or physiological stage) had a significant effect (*p* < 0.01) on BCS evolution with a decrease at 1 week AC in both groups although it was more pronounced in those heifers included in the BCS-3.5 group (0.04 points in BCS-3.5 versus 0.02 points in BCS > 3.5). After delivery and during lactation, the BCS returned to the original values shown at 1 month BC. The BCS index had a significant effect (*p* < 0.01) during the whole study period as the means of the two groups were significantly different. Nevertheless, T*TR was not significant (*p* = 0.710).

Table 2 shows the means for milk production and milk quality in the two groups of cows. The interaction between the BCS group and the time of sampling was not significant for any of the dependent variables. Only the T-factor significantly influenced the milk yield (increased as lactation progressed) and protein percentage (decreased throughout the lactation period) independently of the BCS. Neither the fat percentage, somatic cell count, or F/P ratio were significantly affected by T or BCS.

Table 3 shows the acid–base values throughout the study, as well as significant effects. None of the factors (T, BCS or T*BCS) significantly affected the levels of venous pH or ppCO_2_, and only the T-factor had a significant effect on bicarbonate, BE and TCO_2_, increasing their values after parturition especially at 1 week AC (parallel with the onset of lactation), without differences between the groups.

Table 4 shows the average concentrations of the electrolytes measured, as well as the AG. In this case, the T-factor significantly influenced all parameters, decreasing their concentrations in both groups.

## 4. Discussion

The aim of the study was to obtain a deeper understanding of the physiopathology of the acid–base balance in metabolically burdened dairy heifers with a high body condition in comparison with those in the same physiological situation, although with a BCS in the normal range.

Physiologically, dairy cows lose 0.5 points of the BCS after calving [9]. Increased losses of up to 0.75 points are associated with increased chances of metabolic diseases, such as ketosis. Therefore, minimizing the loss of BCS in the postpartum period (≤0.5 points) is essential for good herd health. Around parturition, dry matter intake (DMI) decreases, resulting in a period of NEB, in which the energy demand for milk synthesis is not covered by voluntary feed intake. To meet the increased energy demands, cows mobilize body reserves predominantly from adipose tissue, promoting metabolic diseases like ketosis. However, high BCS will not necessarily lead to diseases related to metabolic disorders [20]. Obviously, some cows are able to overcome the metabolic adaptation mechanism, as is the case in our study, while others are not [21].

Our results in the farm studied, disagree with the assumptions pointed out by Puppel et al. [8] who indicated that dairy cows with a calving BSC > 3.5, tend to have a risk of ketosis twice as high, compared to those with a BSC of 3.25. As described in later paragraphs, the heifers studied showed no significant differences for either milk production or milk quality, and thus, in ketosis risk. Clearly, the results of the animals in our study, with F/P ratios between 1.17 and 1.29 in the last week AC, were far from existing in an acidotic state caused by the development of ketosis. In addition, as pointed out by Vesna et al. [22], a characteristic of this metabolic stage, deducible from the F/P ratio, is a decrease in milk production, which does not appear in our data.

In our study, we found a positive, but not statistically significant, relationship between BCS and milk production, a fact pointed out by different authors in the literature [23,24,25]. Thus, heifers with a BCS > 3.5 show numerically higher production values one week after calving. In addition, as lactation progressed, the two BCS groups of cows became more similar in their milk production values. There is a certain similarity with the evolution of protein content, attributable to the elimination of colostrum in the first days for both groups, with a decrease at one month AC, without BCS affecting the results.

After the milk production study, and seeing the lack of significant differences between groups, we now address the changes produced in the internal balance, specifically the acid–base equilibrium.

In general terms, throughout the study the animals had a venous pH of 0.01–0.02 points above the one indicated as physiological [26,27], especially after calving, which could imply a tendency towards alkalosis. This is probably due to an increase in the CP intake in the ration (see Table 1), and the significant increase in HCO_3_^−^ levels and BE concentrations may occur as a consequence.

The administration of diets rich in CP during lactation (according to the NRC recommendations of 12% CP for heifers and from 15% to 17% CP for lactating cows) is a very common nutritional management practice. However, in our case, this dietary intake may also have had a buffering effect, acting as a systemic buffer due to ammoniagenesis [28] in response to the well-known increase in metabolic activity characteristic of the onset of lactation [5], which generates an overproduction of H+ as has been previously described [1,29]. In addition, there is a physiological condition that influences the protein content in the bodies of the studied animals: at the start of their first lactation, the competing demands of the mammary gland are superimposed on the requirements for growth.

The increase in both HCO_3_^−^ and BE concentrations is counterbalanced by the increase in TCO_2_, helping to maintain a healthy acid–base balance in blood, and hence, the health status of the animal. This parameter is a measure of CO_2_ that exists in various states: CO_2_ in a physical solution, or slightly bound to proteins, bicarbonate or carbonic acid [26].

There is another fact connected with nutritional management that may have influenced the actions of the buffer systems: after parturition the ration was changed, increasing the content of starch and lowering the content in NDF and ADF. This type of ration, designed to increase milk production, can however, favor ruminal fermentation. This leads to a higher production of ruminal acids [30] that, in turn, are absorbed from the rumen. Thus, under these circumstances, respiratory regulation of carbonic acid in the blood is of particular importance [31].

Serum ions that are included in a chemistry acid–base profile are cations (Na^+^, K^+^) and anions (Cl^−^) and are considered as “fixed ions” because they are bioavailable ions that are not metabolized [32]. As the main extracellular cation responsible for the osmotic force that maintains the extracellular fluid compartment [33], changes in Na^+^ values, especially at 1-month AC, are attributable to the increase in milk production thanks to the lactose synthesized in the udder, which implies free water movement into the udder.

Potassium, as an intracellular ion, is actively involved in cellular metabolism. In the lactating animals under study, the decrease in the values of this electrolyte with respect to the week before parturition is due to the activity of the mammary gland, which requires energy in the form of ATP for the synthesis of lactose, as has been pointed out in several studies [32]. Thanks to the increase in energy provided in the ration through the feed—not through the forage—in this phase, we can understand that although K^+^ levels decrease, they do not reach a point that can be considered pathological. In addition, the “strong ion theory” developed by Stewart and Constable [34] suggests that milk yield and milk composition are not affected by the cation source, with Na^+^ and K^+^ being equally effective [35].

The evolution of Na^+^ and K^+^ ions is related to the systemic generation of HCO_3_^−^. In this line, the Cl^−^ values, which decreased after delivery and as lactation progressed, were the result of a higher excretion as a consequence of a lower systemic generation of H+, as shown by the venous pH values at this stage. In our opinion, the evolution shown by the electrolytes is due to the effect of the increase in CP content during lactation. Many consultants believe that the additional systemic ammonia provided by this type of diet may serve to buffer the high acid loads that occur during milk production, as already pointed out by Cole et al. [28].

Changes in Cl^−^ ion concentrations are closely linked to changes in Na^+^ ions. The decrease in chlorine values after calving, coinciding with those of Na^+^, is due to the free water movement that accompanies milk production and that has already been discussed in previous paragraphs.

The AG represents anions such as Cl^−^, proteins, phosphates, sulfates and organic anions and its increase is associated with situations of metabolic acidosis [33]. Given the lack of data showing that the animals presented acidotic processes, the decrease in the values of this parameter can only be attributed to the effect of the course of time on the electrolytes that generate their value (Na^+^, K^+^, Cl^−^ and HCO_3_^−^), especially the ones that are considered fixed ions [32].

## 5. Conclusions

After analyzing the milk production data and the acid–base data of the heifers in our study, we conclude that the BCS at calving does not affect the productive status during lactation, both in terms of the quantity and quality of milk produced.

The diet provided for each phase did not generate differences between groups. However, we can highlight that the excess of CP in the ration during the lactation phase can trigger a tendency to an alkalotic state, in this case compensated by respiratory buffering mechanisms, as reflected by the TCO_2_. The changes in electrolytes are a reflection of the movement of free water for milk production, in which a balance between measurable anions and cations is observed.

Study limitations: Considering that we were only able to observe a single group of heifers in each of the treatment groups, this study suffers from pseudoreplication and thus the findings of this case study should be viewed with extreme caution. We encourage future work to replicate this work using more groups and ideally under different farm conditions.

## Figures and Tables

**Table 1:** Chemical composition of the TMR fed to the heifers during the present study.

Chemical Composition (g kg^−1^ DM)	BC ^a^	AC
Crude protein (CP)	142.7	171.5
Neutral detergent fibre (NDF)	433.1	307.9
Acid detergent fibre (ADF)	301.2	191.6
Starch	126.8	246.8
Milk forage unit (UFL)	0.76	0.97

^a^ BC, before calving; AC, after calving. All heifers received the same vitamin and mineral premix that changed depending on the studied stage. Thus, BC the composition of the premix was: 28 mg/kg of Cu; 42 mg/kg of Fe; 2.1 mg/kg of I; 126 mg/kg of Zn; 84 mg/kg of Mn; 0.42 mg/kg of Co; 0.63 mg/kg of Se; 16.800 IU vitamin A/kg, 5250 IU vitamin D_3_/kg, 70 IU vitamin E/kg. After calving (AC), the composition was: 12 mg/kg of Cu; 18 mg/kg of Fe; 0.9 mg/kg of I; 54 mg/kg of Zn; 36 mg/kg of Mn; 0.180 mg/kg of Co; 0.27 mg/kg of Se; 7.200 IU vitamin A/kg, 2250 IU vitamin D_3_/kg, 30 IU vitamin E/kg.

**Table 2:** Mean values (±standard error of the mean, SEM) of productive parameters depending on BCS group and their interactions during the study period.

Parameter	Group ^a^	Samplings		*p*-Value		
		1 Week AC ^b^	1 Month AC	T	BCS	T*BCS
Milk yield	BCS < 3.5	34.1 ± 3.5	41.5 ± 3.5	0.026	0.638	0.609
Kg	BCS > 3.5	37.1 ± 2.4	41.9 ± 2.4			
Fat	BCS < 3.5	4.12 ± 0.30	4.26 ± 1.53	0.495	0.941	0.168
(%)	BCS > 3.5	4.42 ± 0.30	4.01 ± 0.20			
Protein	BCS < 3.5	3.52 ± 0.13	3.32 ± 0.09	0.016	0.400	0.722
(%)	BCS > 3.5	3.44 ± 0.13	3.19 ± 0.09			
F/P ratio	BCS < 3.5	1.17 ± 0.08	1.28 ± 0.05	0.483	0.519	0.242
	BCS > 3.5	1.29 ± 0.08	1.26 ± 0.05			
SCCn ^c^	BCS <- 3.5	2.87 ± 0.57	2.33 ± 0.39	0.088	0.659	0.623
	BCS > 3.5	2.82 ± 0.57	1.88 ± 0.39			

^a^ BCS: Body Condition Score; ^b^ AC: after calving; ^c^ SCCn Somatic cell concentration.

**Table 3:** Mean values (±standard error of the mean, SEM) of acid–base parameters depending on BCS group during the study period.

Parameter	Group ^a^	Samplings				*p*-Value		
		1 Month BC ^b^	1 Week BC	1 Week AC ^c^	1 Month AC	T	BCS	T*BCS
Venous	BCS < 3.5	7.41 ± 0.01	7.43 ± 0.02	7.44 ± 0.01	7.44 ± 0.01	0.085	0.594	0.836
pH	BCS > 3.5	7.42 ± 0.01	7.44 ± 0.01	7.44 ± 0.01	7.44 ± 0.01			
pCO_2_	BCS < 3.5	43.8 ± 1.92	40.8 ± 2.25	44.2 ± 0.01 ± 1.16	43.7 ± 1.07	0.073	0.809	0.868
(mm Hg)	BCS > 3.5	42.3 ± 0.93	40.6 ± 0.87	45.0 ± 1.28	43.4 ± 1.36			
HCO_3_^−^	BCS < 3.5	27.7 ± 1.53	27.0 ± 0.96	30.3 ± 2.02	29.6 ± 2.05	<0.001	0.899	0.919
(mmol/L)	BCS > 3.5	27.3 ± 1.85	27.7 ± 1.93	30.4 ± 1.43	29.5 ± 1.68			
BE ^d^	BCS < 3.5	3.2 ± 1.03	3.0 ± 1.19	6.4 ± 0.62	5.4 ± 0.54	<0.001	0.941	0.937
(mmol/L)	BCS > 3.5	2.8 ± 0.50	3.6 ± 0.46	6.2 ± 0.69	5.2 ± 0.73			
TCO_2_	BCS < 3.5	29.0 ± 1.77	28.0 ± 2.05	31.8 ± 1.07	30.8 ± 0.98	0.32	0.396	0.370
(mmol/L)	BCS > 3.5	28.5 ± 0.86	28.8 ± 0.79	31.7 ± 1.18	27.5 ± 1.25			

^a^ BCS: Body Condition Score; ^b^ BC: before calving; ^c^ AC: after calving; ^d^ BE: Base excess.

**Table 4:** Mean values (± standard error of the mean, SEM) of blood electrolytes depending on BCS group, time of sampling, and their interactions during the study period.

Parameter	Group ^a^	Samplings				*p*-Value		
		1 Month BC ^b^	1 Week BC	1 Week AC ^c^	1 Month AC	T	BCS	T*BCS
Na+	BCS < 3.5	142.2 ± 0.9	143.0 ± 1.0	142.4 ± 0.5	139.9 ± 0.5	<0.001	0.933	0.705
(mmol/l)	BCS > 3.5	142.7 ± 0.4	143.7 ± 0.5	141.7 ± 0.6	139.6 ± 0.6			
K^+^	BCS < 3.5	3.8 ± 0.1	4.5 ± 0.2	3.5 ± 0.1	3.5 ± 0.08	<0.001	0.403	0.235
(mm Hg)	BCS > 3.5	3.8 ± 0.1	3.9 ± 0.1	3.5 ± 0.1	3.6 ± 0.1			
Cl^−^	BCS < 3.5	100.7 ± 1.2	103.3 ± 1.4	100.6 ± 0.7	98.4 ± 0.7	<0.001	0.568	0.493
(mmol/L)	BCS > 3.5	101.8 ± 0.6	103.4 ± 0.5	99.7 ± 0.8	99.6 ± 0.9			
AG ^d^	BCS < 3.5	17.5 ± 0.9	16.7 ± 1.0	14.8 ± 0.5	15.4 ± 0.5	<0.001	0.435	0.580
(mmol/L)	BCS > 3.5	17.2 ± 0.4	16.5 ± 0.4	15.1 ± 0.6	14.1 ± 0.6			

^a^ BCS: Body Condition Score; ^b^ BC: before calving; ^c^ AC: after calving; ^d^ AG: Anion gap.

## Data Availability

Not applicable.
